# Trends in Well-Child Visits and Routine Vaccination among Children of U.S. Military Members: An Evaluation of the COVID-19 Pandemic Effects

**DOI:** 10.3390/jcm11226842

**Published:** 2022-11-19

**Authors:** Kyle Sexton, Apryl Susi, Elizabeth Lee, Elizabeth Hisle-Gorman, Michael Rajnik, Jayasree Krishnamurthy, Cade M. Nylund

**Affiliations:** Department of Pediatrics, F. Edward Hebert School of Medicine, Uniformed Services University of the Health Sciences, Bethesda, MD 20814, USA

**Keywords:** COVID-19, well-child visits, pediatric vaccination, military dependents, health services utilization

## Abstract

The COVID-19 pandemic has drastically impacted administration of healthcare including well-child visits and routine vaccinations. The purpose of this study was to determine the impact of COVID-19 pandemic disruption on childhood health maintenance: well-child visits and scheduled vaccinations. We queried the TRICARE Management Activity’s Military Health System (MHS) database for outpatient well-child visits and vaccinations for all children 0 to 23 months of age eligible for TRICARE healthcare. The median rate of well-child visits, during the COVID-19 period (March 2020–July 2021), was significantly declined for all demographic groups: all ages, parental military ranks, sex, and regions as compared to the pre-COVID-19 period (February 2019–February 2020). Similar to rates of well-child visits, the rate of vaccinations declined during the COVID-19 period as compared to the pre-COVID-19 period for all demographic groups, except children 12–23 months. Rates of well-child visits for military dependent children under 2 years of age were decreased during the 16 month COVID-19 period, with large increases seen in the first 2 months of the pandemic; the consequences of missed well-child visits and vaccination are unknown.

## 1. Introduction

The COVID-19 pandemic has drastically impacted the administration of healthcare globally and in the United States (U.S.). The U.S. Military Health System (MHS), an extensive system of care delivered at both military and civilian facilities to TRICARE insurance beneficiaries, was not immune to these impacts. Beginning in March 2020, efforts to reduce transmission and decreased capacity due to a focus on COVID care led to postponement of non-urgent medical surgeries and treatments, reductions in available appointments, and documented declines in adult preventive healthcare visits [1,2,3]. Early indicators also suggested drops in well-child visits and vaccination rates for children under 18 [4,5].

The American Academy of Pediatrics’ (AAP) goals of well-child visits include preventive medical counseling, tracking childhood health and development, allowing parents to raise health concerns, and facilitating a team approach to early childhood healthcare [4,5,6,7]. The first two years of life have a regimented schedule of well-child visits, strategically planned for optimized timing of vaccinations, screening for congenital disease, proper growth and developmental disorders [6]. During this timeframe, the AAP and U.S. Centers for Disease Control and Prevention (CDC) recommend a minimum of ten well-child visits and nine different sets of vaccines (excluding yearly influenza). Well-child care is recommended to be delivered by a primary physician who assumes responsibility for the patient’s care and can include team based care provided by other appropriately licensed healthcare providers [8]. 

The well-child visit and vaccination schedule is integral to the health of an infant to prevent as well as facilitate early diagnosis of morbidity and mortality. In the MHS, pediatric beneficiaries receive well-child services and routine immunizations at pediatric clinics in military or civilian settings that accept TRICARE insurance and are staffed by pediatricians and nurses. Prior to the COVID-19 pandemic, children 0 to 15 months of age adhered to the established schedule of six visits 86% of the time, with a mean visit frequency of 6.7 well child visits through 15 months of age [9]. By comparison, studies of U.S. children with a different insurance mix receiving care outside of the MHS suggest adherence may be somewhat lower, at 83.2% in children <12 months of age and 68.1% in children 12 to 23 months, and that this may be highly context- and population-dependent [10,11].

Healthcare disparities in well-child care and early life vaccination have been documented in the pre-COVID-19 period. Children who live in rural areas, live in lower income families, and have older parents tend to attend fewer well-child appointments [12,13]. Within the military, previous research indicates that children of lower ranking parents, younger parents, and those living in the central region of the U.S. or overseas had lower rates of well-child care 2014–2016. Understanding the impact of demographic factors during the COVID-19 period may be important to provide targeted intervention to those groups with the largest decreases in care.

Although U.S. military beneficiary healthcare coverage is universal, pediatric beneficiaries may have unique challenges in adherence to well-child visits. For example, variable demographic characteristics such as geographic location (urban, remote or overseas), rank of sponsor as a proxy for socioeconomic status, and parent’s permanent change of station frequency may affect adherence to the standard visit schedule [9]. The effect of the COVID-19 pandemic on these risk factors is unknown. To elucidate this relationship, we evaluate the effect of the COVID-19 time period on well-child visit and vaccination rates for children under two, and explore heterogeneities in rates by military-relevant demographic factors.

## 2. Materials and Methods

We performed a retrospective repeated monthly cross-sectional study using data from the TRICARE Management Activity’s MHS database. TRICARE is the Department of Defense’s health care program for members of the uniformed services and their families. The MHS database records TRICARE billing data for outpatient and inpatient encounters of beneficiaries from both civilian and military treatment facilities. These beneficiaries include children within a broad spectrum of socioeconomic classes whose parents are stationed at military locations throughout the US and the world. We queried the MHS database for outpatient well-child visits and vaccinations for all children 0 to 23 months of age eligible for TRICARE healthcare.

U.S. uniformed services’ beneficiaries aged 0 to <24 months who were enrolled in TRICARE in a given month from February 2019 to July 2021 were eligible for inclusion; this number fluctuated over the course of the study based on age inclusion criteria per given month. Well-child visits were counted monthly and identified by clinical encounters marked with either corresponding International Classification of Diseases (ICD) codes or Current Procedural Terminology (CPT) codes indicative of well-child care (Supplemental Table S1). All visits for individuals 24 months or older at the time of the visit were excluded. Vaccinations for hepatitis B, rotavirus, diphtheria, tetanus, pertussis, *Haemophilus influenzae* type B, pneumococcus, polio, measles, mumps, rubella, varicella, hepatitis A were summed monthly and identified via CPT codes (Supplemental Table S2). All of the above vaccinations received from an institution (public health clinic, pharmacy, medical center, etc.) that accepts TRICARE insurance and given in the appropriate study inclusion criteria were included. Influenza vaccination was not included due to seasonal use. With performing monthly cross-sectional data, we would expect a seasonal increase in the fall and early winter if influenza was included. The Pre-COVID-19 and COVID-19 periods contain unequal months and seasons for which influenza vaccine was excluded. We calculated monthly rates of well-child visits and vaccinations by dividing the total number of well-child visits or vaccinations in children <24 months given in a month by the number of children <24 months enrolled and eligible for care in that same month. 

Child’s sex, age, military parent (or sponsor) rank, and geographic region of care were extracted from the monthly enrollment records. Child age was grouped into 0–11 months if the child was <12 months of age, and 12–23 months if the child was between 12 months to <24 months of age in a given month. Sponsor rank was categorized as junior enlisted (JrEn), senior enlisted (SrEn), or officer rank. Junior enlisted rank can serve as an effective surrogate for lower parental education and lower income, as junior enlisted service members generally do not have more than a high school education, and generally earn less than $30,000 per year [14,15]. Geographic region of care is based on the TRICARE definitions of North, South, West, and outside of the continental U.S., which includes overseas locations in addition to Alaska and Hawaii. Time period was defined as pre-COVID-19 (February 2019 to February 2020) and COVID-19 period (March 2020–July 2021). The pre-COVID-19 and COVID-19 period lengths are asymmetric in length because the initial protocol was written early in the COVID-19 pandemic to collect data one year before the start of COVID-19 and the COVID-19 affected period has persisted longer than expected.

Due to the varying nature of enrollment in electronic health records data and non-normal distributions of the data, we calculated the median percent of the monthly patient population in each demographic category. We compared results for the COVID-19 and pre-COVID-19 time periods using Wilcoxon Rank sum two-sided z-tests. Medians of monthly well-child visits and vaccination rates were compared across the time periods as well for each demographic group using the same methodology.

We used unadjusted Poisson regression to estimate rate ratios for well-child visit and vaccination rates by time period, age, sex, parent rank, and geographic region, respectively, controlling for variation in monthly enrollment through an offset term. Because it was hypothesized that the relationships observed between groups may differ over the time periods, interactions between time period and demographic variables were tested to examine whether there were significant changes in demographic variables during the study period. When interactions existed the results were stratified by time period and the adjusted models are presented. As a consequence of changing demographics over the study period, we are unable to present a single adjusted rate ratio (RR) comparing the COVID-19 period to the pre-COVID-19 period. We do calculate the rate ratios for time period for each unique demographic combination of the variables with significant interactions.

## 3. Results

There were a total of 524,384 individual children included over the study period. Median monthly enrollment over the study period was 220,144 children <24 months of age (IQR: 216,605–222,999). Monthly enrollment varied from a high of 224,618 in May 2019 to a low of 210,447 in May 2021. Our population was slightly more male and older (12–23 months) for the study period (Table 1). Over half had a senior enlisted parent, and most were geographically dispersed evenly across north, south and west regions, with the remaining 7% receiving care outside the continental U.S. We found statistically significant differences for within-group median monthly percent of all population demographic characteristics when comparing the pre- and COVID-19 time periods, with the exception of children of junior enlisted and senior enlisted rank (Table 1). However, these differences may not be meaningful due to the size of our study population.

### 3.1. Well-Child Visits in the Pre-COVID-19 and COVID-19 Time Periods

**Figure 1:**
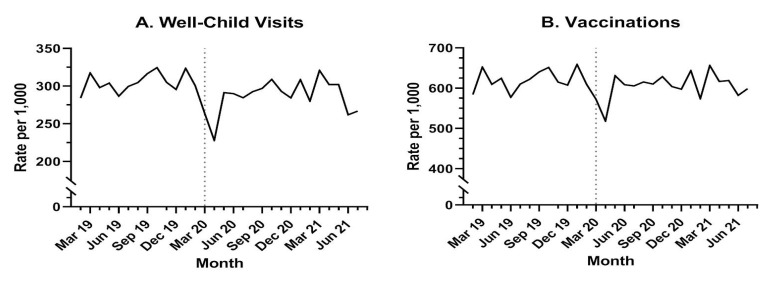
Well-Child Visit and Vaccination Rates: (**A**) Rates of well-child visits are reported per 1000 enrollees under 24 months of age; (**B**) rates of vaccination are reported per 1000 enrollees under 24 months of age.

**Figure 2:**
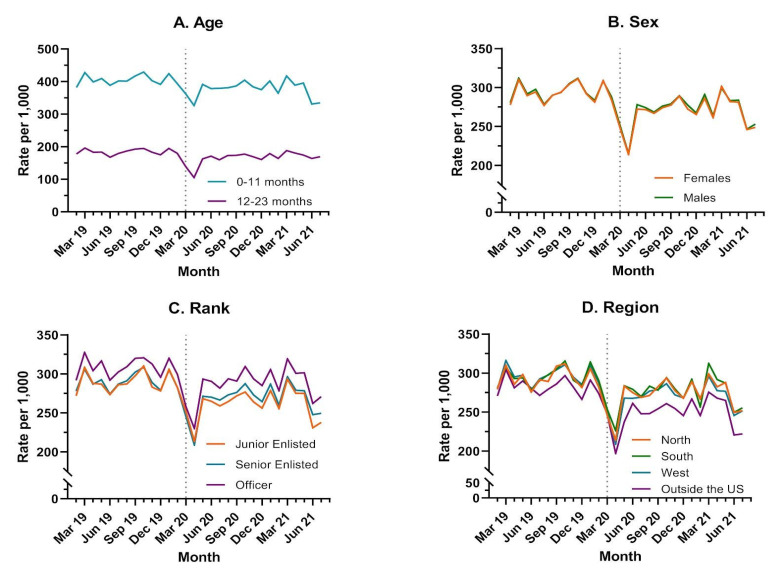
Well-Child Visit Rate by Demographic Category: Rates of well-child visits are reported per 1000 enrollees under 24 months of age by (**A**) Age by (**B**) Sex by (**C**) Rank and by (**D**) Geographic Region of the Military Health System.

Overall, the median well-child visit rate per 1000 children <24 months for the study period was 282 (IQR: 273–292) (Table 2). There was a statistically significant difference (*p* < 0.0001) in median monthly visit rates for the overall study population when comparing the pre-COVID-19 (291, IQR: 282–305) and COVID-19 (275, IQR: 264–283) periods. Despite general variability over the entire study period, a noticeable drop in the rate of well-child visits occurred in March 2020 (Figure 1a). The median rate of well-child visits, when compared for the pre-COVID-19 versus COVID-19 periods, significantly declined for all demographic groups: all ages, parental military ranks, regions and both sexes (Table 2). The largest absolute declines in median rates occurred in children 0–11 months, those with a junior enlisted parent, and in the West and outside of the continental U.S. (Table 2). In general, children 0–11 months, as well as those with an officer ranked-parent, had higher rates of well-child visits over time, whereas those receiving care outside the continental U.S. had significantly lower rates compared to other regions (Figure 2 and Table 2). We found significant interactions between time period (pre-COVID-19 and COVID-19) and age, rank and region, respectively (Table 3 and Table 4). However, there was no discernable difference in well-child visit rate by sex over time (*p* = 0.9771) (Table 3 and Figure 2).

In Table 4, we are unable to present a single rate ratio comparing the COVID-19 period to the pre-COVID period, but across all demographic groups there was a statistically significant decrease in the rate of well child visits. The decrease was most noticeable again in the other/overseas population, and was more pronounced in the 1 year olds than the 0 year olds.

### 3.2. Vaccination in the Pre-COVID-19 and COVID-19 Time Periods

**Figure 3:**
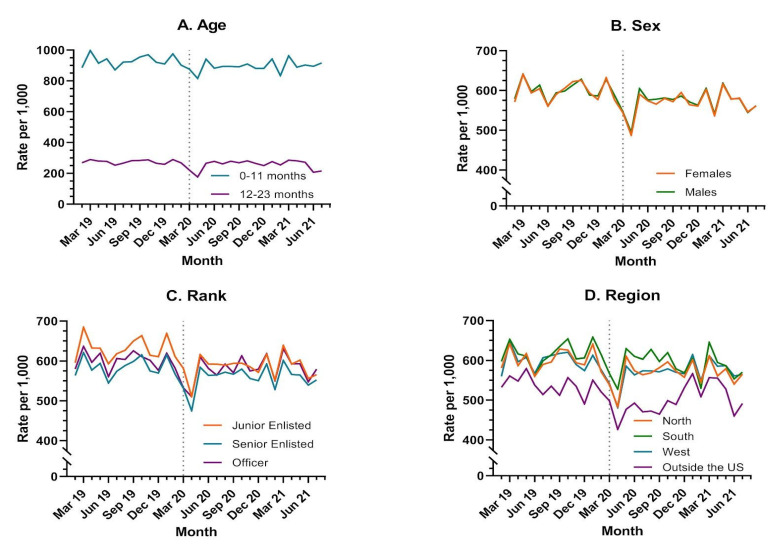
Vaccination Rates by Demographic Category: Rates of the administration of vaccines in children less than 24 months are reported per 1000 enrollees by (**A**) Age by (**B**) Sex by (**C**) Rank and by (**D**) Geographic Region of the Military Health System.

The overall median vaccination rate per 1000 children <24 months of age was 581 vaccines administered per month (IQR: 568–602) for our study period (Table 2). There was a statistically significant difference (*p* < 0.0001) in median monthly vaccination rates for the overall study population when comparing the pre-COVID-19 (594, IQR: 582–618) and COVID-19 (575, IQR: 562–586) periods. In line with well-child visits, a decline in the overall vaccination rate occurred between January–March 2020, (Figure 1b). Similar to rates of well-child visits, the median rate of vaccinations declined in the COVID-19 period as compared to the pre-COVID-19 period for all demographic groups, although for children 12–23 months this decline was nonsignificant (*p* = 0.0542) (Table 2). As with well-child visits, the largest absolute declines in median vaccination rates occurred in children 0–11 months, those with a junior enlisted parent, and those in the west and outside of the continental U.S. as compared to the south and the north. In general, children 0–11 months of age had higher rates of vaccination over time as compared to those 12–23 months of age, whereas those with a senior enlisted ranked-parent as well as those receiving care outside the continental U.S. had lower rates of vaccination compared to other ranks and regions, respectively (Figure 3 and Table 2). For vaccination rates, there were significant interactions between time period and age, parent rank, and region outside the continental U.S. versus west (all *p* < 0.0001) (Table 5 and Table 6). Over pre- and COVID-19 time periods, there were no differences in vaccination rates by sex (*p* = 0.5036), north versus west (*p* = 0.1396), or south versus west (*p* = 0.1894) regions (Table 5 and Figure 3). 

For Table 6, we are not able to obtain a single rate ratio for comparing the COVID-19 period vs. pre-COVID-19 periods; there was a statistically significant decrease in the rate of vaccinations across all of the demographic groups, with those living outside of the US experiencing the greatest declines.

## 4. Discussion

In this analysis of the effect of the COVID-19 pandemic on preventive health service utilization for children under two years of age in the military health system, we illustrate declines in rates of well-child visits and vaccine administration during the COVID-19 time period for all demographic groups studied. The initial drop in March–April 2020 in both well-child visits and vaccinations coincided with the beginning of the pandemic lock-down and social distancing mandates, with rates rebounding in May-June, but the overall COVID-19 period rate was lower than the pre-COVID-19 period.

This was observed most significantly in the junior enlisted population, western region, and outside the U.S. region. Potential explanations for the more pronounced decrease in well-child visit rates for junior enlisted include decreased capacity for health advocacy during a period of difficulty in accessing care, apprehension of the medical system for risk of exposure to COVID-19, and decreased access to care at smaller health care centers [16]. Most commonly, U.S. military service members can be located at bases that are smaller with resources that were most likely at capacity during COVID-19 restrictions. Despite pronounced decreases in well-child care for children of junior enlisted parents, and decreased vaccination rates in this group during COVID-19, the monthly vaccination rate for junior enlisted (as shown in Figure 3c) was higher than the rate for senior enlisted or officer children. Children of junior enlisted had lower rates of well-child visits but were able to obtain vaccinations at a higher rate than other senior ranks. These results suggest that junior enlisted parents may be more likely to comply with vaccine guidance despite missing well-child encounters, or that they were only able to access alternative vaccination without a combined well-child visit. Additionally, this trend is important to identify junior enlisted as a high risk group for decreased health maintenance of beneficiaries under two years old. The analysis of the demographics showed no significant difference of well-child visit rates or vaccinations between and male vs. female. This further demonstrates that the impact of rank and region effects on the health maintenance rates and provides an area of further study to determine health outcomes due to missed preventive care and to seek system changes to facilitate junior rank military member’s access and preventive healthcare for their children.

Following the onset of the pandemic and the closure of most routine services with stay at home orders in March 2020, access to routine healthcare became encumbered. Vaccination and well-child visit rates effects from the COVID-19 pandemic have been observed to drop in the first months of the pandemic [4,5]. The literature is inconsistent regarding the pandemics disruption, however, in the above studies, rates appear to have normalized by July 2020 and continued through June 2021 in their local civilian study populations. Studies have shown that ages zero to one had limited changes to vaccination and well-child visits even with the impact of COVID-19 [1,5]. This is one major difference in our study which saw a significant decrease in the median rates of well-child visits and vaccinations during the COVID-19 pandemic for children aged <1 year of age. Our study is limited to military dependents and covers a wide breadth of geographical area which may explain the resultant differences from previous studies.

Our study population are military dependents under two years of age. The military is a very diverse population that closely reflects the demographics of the U.S. population [15]. Unlike the U.S. population, all service members have the same access to high quality healthcare, from the youngest enlisted family which may fall under the poverty line, to the highest paid officer. In this study, despite access to healthcare, rank(income) appears to impact the use of well-child care during the pandemic, with higher paid (and higher ranking) individuals accessing more preventive care for their children, but not accessing more vaccination. 

Reflecting upon the beginning of the pandemic for our healthcare system, it is not at all surprising that for 2 months at the beginning of the COVID-19 pandemic we saw a substantial decrease across geographical regions in the healthcare services (well-child visits and immunizations). Regional variations in the delivery of healthcare during the COVID-19 pandemic were observed. In particular, a larger sustained decline was noted in the West and outside the continental U.S. regions. This variability may be able to be explained by regional variations such as state or country restrictions of movement that existed in the pandemic period. For instance, the western state of California, which has a large number of military beneficiaries, had some of the longest and most heavily adhered to social distancing mandates in the United States. Internationally, U.S. bases in countries like the Republic of Korea and Japan operated under vastly different public health rules than the U.S. and may have made access to healthcare more difficult, particularly for purchased care outside of the military health system.

Restrictions of movement and variation of health administration during the lockdowns lead to several different modalities which were initiated to attempt to increase health maintenance. One modality that may have contributed to rebound in health maintenance is the broader use of telehealth and virtual encounters. By March 17, 2020 CMS (Centers for Medicare and Medicaid Services) had expanded the use of telehealth for older adults allowing for equal reimbursement to face-to-face encounters [17,18,19]. This led to an increased acceptance of telehealth by other insurers and a rapid expansion of virtual healthcare visits as the technology became available.

Novel delivery of immunizations is another modality that was initiated to maintain vaccination schedules among this population. While typically tied to well-child visits, immunizations for children began to be distributed in many novel ways. As healthcare systems developed drive through testing sites, they also began to use similar techniques to mass deliver immunizations and other healthcare [20]. Patients began to rely more readily on county and state public health immunization offerings that may not be captured by our analysis of our available data. Pharmacists began giving immunizations down to the age of 3 as part of the Public Readiness and Emergency Preparedness Act in August of 2020 with permanent expansion in December 2020 [21]. 

In Europe, the military bases and their healthcare facilities operated based on the rules of the host country. Most healthcare was not delivered in the spring and summer of 2020. For military members and their families assigned overseas, there are very few alternatives to receive primary care other than the military treatment facilities. Furthermore, the healthcare system overseas did not have the capacity to surge in order to catch up on missed appointments when healthcare became more available. Since our study only included children up to the age of 24 months, it is possible that children are continuing to receive catch up immunizations beyond 24 months of age which were undetected by our study.

In this study, rates of well-child visits and vaccinations continued to be below pre-COVID-19 levels. If the rates continue to be decreased, this could pose several potential problems with general health maintenance in children under 2 years old. Missed well-child appointments could lead to delay in the diagnosis of growth disorders or developmental disorders such as autism. Decreased vaccination could lead to a rise in rates of preventable infectious diseases to include measles, mumps, pertussis, etc. This is an area of study that needs continued research to determine if well-child and vaccinations rates increased after July 2021 and whether there have been adverse outcomes as a result of missed well-child visits.

Our study is subject to a few limitations. We are not able to conclude on the direct cause of the decline in well-child visits, i.e., if patients’ perceptions of the health risks of visiting a physician versus institutional limitations in services due to transmission precautions. We also did not evaluate normal seasonality of vaccine administration. Another limitation is that this information may not be generalizable to the U.S. population as the dependents of military members have robust access to insurance and medical facilities in most regions of the country. Lastly, among the military population, vaccinations and well-child visits were only included if TRICARE insurance was accepted and billed for both military and civilian medical facilities.

## 5. Conclusions

Rates of well-child visits in military dependent children under 2 years of age decreased substantially in the first 2 months of the pandemic, and sustained a significant decrease throughout the COVID-19 period. This was most apparent in our junior enlisted population and those assigned overseas. Due to this decline, further investigation into missed well-child visits and vaccination especially in the junior enlisted population and other lower economic status groups should be explored to find approaches to remove barriers to accessing care now and during unforeseen future disruptions in healthcare delivery.

## Figures and Tables

**Table 1:** Population demographics as the median monthly percent of population within each demographic group.

	Full Study Period Median (IQR)	Pre-COVID-19 Median (IQR)	COVID-19 Period Median (IQR)	*p*-Value
Age in months				
0–11 months	49.36 (49.16–49.70)	49.73 (49.69–49.77)	49.18 (49.15–49.25)	<0.0001
12–23 months	50.64 (50.30–50.84)	50.27 (50.23–50.31)	50.82 (50.75–50.85)	<0.0001
Parent’s military rank				
Junior Enlisted	26.59 (26.52–26.76)	26.68 (26.53–26.76)	26.59 (26.44–26.74)	0.4513
Senior Enlisted	52.72 (52.58–52.84)	52.78 (52.66–52.85)	52.67 (52.53–52.80)	0.1672
Officer	20.68 (20.60–20.75)	20.59 (20.55–20.62)	20.73 (20.69–20.82)	<0.0001
Sex				
Female	48.82 (48.76–48.87)	48.67 (48.86–48.88)	48.77 (48.70–48.83)	0.0011
Male	51.18 (51.13–51.24)	51.13 (51.12–51.14)	51.23 (51.17–51.30)	0.0011
Region				
North	30.58 (30.54–30.69)	30.70 (30.61–30.74)	30.55 (30.52–30.58)	0.0003
South	29.38 (29.19–29.49)	29.19 (29.17–29.22)	29.48 (29.42–29.53)	<0.0001
West	32.94 (32.83–33.03)	32.83 (32.79–32.87)	33.00 (32.92–33.10)	0.0003
Outside the continental U.S.	7.18 (6.94–7.27)	7.28 (7.27–7.31)	6.96 (6.85–7.10)	<0.0001

**Table 2:** Pre-COVID-19 versus COVID-19 Period: Median Monthly Well-Child Visit and Vaccination Rates.

	Well-Child Visit Rate per 1000 Children <24 Months				Vaccination Rate per 1000 Children <24 Months			
	Full Study Median (IQR)	Pre-COVID-19 Median (IQR)	COVID-19 Median (IQR)	*p*-Value	Full Study Median (IQR)	Pre-COVID-19 Median (IQR)	COVID-19 Median (IQR)	*p*-Value
Overall Population								
All	282 (273–292)	291 (282–305)	275 (264–283)	<0.0001	581 (568–602)	594 (582–618)	575 (562–586)	<0.0001
Age in months								
0–11	391 (379–403)	402 (394–417)	381 (364–391)	0.0013	906 (885–941)	922 (910–954)	894 (882–909)	0.0135
12–23	176 (168–183)	183 (179–193)	169 (163–174)	0.0004	268 (259–281)	278 (266–284)	265 (251–278)	0.0542
Parent Rank								
Junior Enlisted	275 (264–287)	287 (283–298)	264 (255–275)	0.0001	599 (590–627)	627 (611–650)	592 (572–595)	0.0003
Senior Enlisted	279 (270–289)	289 (282–302)	271 (260–279)	0.0004	569 (556–589)	577 (570–599)	565 (550–572)	0.0107
Officer	298 (291–310)	309 (299–320)	291 (278–301)	0.0020	592 (575–610)	604 (582–619)	580 (565–593)	0.0364
Sex								
Females	281 (272–292)	292 (284–304)	272 (261–281)	0.0003	579 (564–603)	594 (577–622)	572 (561–581)	0.0095
Males	283 (274–293)	293 (288–305)	276 (264–283)	0.0003	584 (571–546)	597 (588–614)	578 (562–581)	0.0039
Region								
North	283 (271–294)	292 (282–306)	275 (267–283)	0.0034	581 (563–601)	594 (587–626)	569 (558–582)	0.0065
South	286 (278–294)	293 (290–305)	279 (257–287)	0.0044	604 (579–619)	614 (604–635)	595 (569–610)	0.0265
West	279 (268–295)	295 (288–304)	269 (267–278)	<0.0001	578 (566–608)	606 (576–613)	571 (563–585)	0.0074
Outside the continental U.S.	267 (248–281)	281 (274–290)	248 (245–261)	<0.0001	517 (490–548)	535 (521–550)	493 (472–528)	0.0065

**Table 3:** Well-Child Visit Rate Ratios (RR).

	Unadjusted RR (95% CI)	Pre-COVID-19 Period Adjusted RR (95% CI)	COVID-19 Period Adjusted RR (95% CI)
Age 12–23 months vs. 0–11 months *	0.45 (0.44–0.45)	0.45 (0.45–0.45)	0.44 (0.44–0.44)
Rank Officer vs. Junior Enlisted *	1.08 (1.08–1.09)	1.11 (1.10–1.11)	1.14 (1.13–1.14)
Rank Senior Enlisted vs. Junior Enlisted *	1.01 (1.01–1.02)	1.05 (1.05–1.06)	1.07 (1.06–1.08)
Sex Female vs. Male	0.99 (0.99–0.996)	0.99 (0.99–0.997)	0.99 (0.99–0.997)
Region North vs. West *	1.00 (0.999–1.01)	1.00 (0.99–1.001)	1.02 (1.02–1.03)
Region Outside Continental U.S. vs. West *	0.94 (0.94–0.95)	0.95 (0.94–0.96)	0.92 (0.91–0.92)
Region South vs. West *	1.01 (1.01–1.02)	1.01 (1.004–1.02)	1.04 (1.03–1.04)
Time Period COVID-19 vs. Pre-COVID-19	0.93 (0.93–0.93)	—	—

* *p*-value < 0.05 for significant interaction with time period.

**Table 4:** COVID-19 versus Pre-COVID-19 Period Rate Ratios for Well-Child Visits Stratified by Demographic Subsets.

Age	Rank	Region			
		North	South	West	Outside the Continental U.S.
0–11 months	Junior Enlisted	0.93 (0.92–0.94)	0.93 (0.92–0.95)	0.93 (0.91–0.94)	0.89 (0.87–0.92)
	Senior Enlisted	0.95 (0.94–0.95)	0.95 (0.94–0.96)	0.93 (0.92–0.94)	0.91 (0.89–0.92)
	Officer	0.96 (0.95–0.97)	0.95 (0.94–0.96)	0.95 (0.93–0.96)	0.92 (0.90–0.95)
12–23 months	Junior Enlisted	0.92 (0.90–0.93)	0.94 (0.92–0.95)	0.87 (0.85–0.89)	0.85 (0.81–0.89)
	Senior Enlisted	0.94 (0.93–0.96)	0.94 (0.93–0.95)	0.90 (0.89–0.91)	0.84 (0.82–0.86)
	Officer	0.96 (0.95–0.98)	0.96 (0.94–0.98)	0.91 (0.89–0.92)	0.86 (0.82–0.89)

**Table 5:** Vaccination Rate Ratios.

	Unadjusted RR (95% CI)	Pre-COVID-19 Period Adjusted RR (95% CI)	COVID-19 Period Adjusted RR (95% CI)
Age 12–23 months vs. 0–11 months *	0.29 (0.29–0.29)	0.29 (0.29–0.29)	0.29 (0.29–0.29)
Rank Officer vs. Junior Enlisted *	0.97 (0.97–0.98)	1.005 (1.001–1.009)	1.04 (1.04–1.05)
Rank Senior Enlisted vs. Junior Enlisted *	0.94 (0.94–0.94)	0.99 (0.99–0.997)	1.02 (1.01–1.02)
Sex Female vs. Male	0.996 (0.994–0.998)	0.995 (0.992–0.998)	0.996 (0.994–0.999)
Region North vs. West	1.00 (0.998–1.003)	1.02 (1.01–1.02)	1.01 (1.01–1.02)
Region Outside the Continental U.S. vs. West *	0.89 (0.88–0.89)	0.89 (0.89–0.90)	0.87 (0.86–0.87)
Region South vs. West	1.03 (1.03–1.04)	1.05 (1.04–1.05)	1.05 (1.05–1.06)
Time Period COVID-19 vs. Pre-COVID-19	0.96 (0.96–0.96)	—	—

* *p*-value < 0.05 for significant interaction with time period.

**Table 6:** COVID-19 versus Pre-COVID-19 Period Rate Ratios for Vaccinations Stratified by Demographic Subsets.

Age	Rank	Region			
		North	South	West	Outside the Continental U.S.
0–11 months	Junior Enlisted	0.95 (0.95–0.96)	0.96 (0.95–0.96)	0.95 (0.95–0.96)	0.91 (0.89–0.93)
	Senior Enlisted	0.96 (0.96–0.97)	0.98 (0.98–0.99)	0.97 (0.96–0.97)	0.95 (0.94–0.97)
	Officer	0.98 (0.97–0.99)	0.98 (0.97–0.99)	0.99 (0.98–0.999)	0.95 (0.94–0.97)
12–23 months	Junior Enlisted	0.93 (0.92–0.95)	0.91 (0.89–0.92)	0.92 (0.91–0.94)	0.88 (0.85–0.92)
	Senior Enlisted	0.95 (0.94–0.96)	0.96 (0.95–0.97)	0.96 (0.95–0.97)	0.92 (0.90–0.94)
	Officer	0.98 (0.96–0.99)	0.97 (0.95–0.98)	0.96 (0.95–0.98)	0.95 (0.91–0.99)

## Data Availability

All data were accessed from the Military Health System database, which requires a valid data sharing agreement due to the nature of personal health information and cannot be shared without period approval from the US Department of Defense, Defense Health Agency.

## Supplementary Materials

The following supporting information can be downloaded at: https://www.mdpi.com/article/10.3390/jcm11226842/s1, Supplemental Table S1: Well-Child Visit ICD and CPT codes; Supplemental Table S2: Vaccination CPT Codes.
