# Supplementary material for: Trends in Well-Child Visits and Routine Vaccination among Children of U.S. Military Members: An Evaluation of the COVID-19 Pandemic Effects
Source: J Clin Med. 2022 Nov 19;11(22):6842. doi: 10.3390/jcm11226842 (PMC9699213; doi:10.3390/jcm11226842)
Supplement: Supplementary file 1 [file jcm-11-06842-s001.zip › jcm-1986816-supplementary.pdf]

Supplemental Table S1: Well-Child Visit ICD and CPT codes.

Z00.01	Encounter for general adult medical examination with abnormal findings
Z00.110	examination under 8 days old
Z00.111	examination 8 to 28 days old; weight check
Z00.121	examination over 28 days old with abnormal finding
Z00.129	examination of infant or child over 28 days
Z00.20	routine examination for period of rapid growth
Z00.30	evaluation for developmental state of adolescence
Z02.5	examination for sports
Z76.1	healthy infant or child
Z76.2	well-baby- well-child

CPT Codes	Description
99381	The provider performs a well–baby visit for a child under the age of one.
99382	The provider performs a well–patient visit for a child who is between the ages of 1 and 4 who meets the definition of new patient.
99391	Preventive medicine services are provided to individuals who are not in need of any immediate medical attention but need to undergo a periodic health checkup as a preventive measure.
99392	Preventive medicine services are provided to individuals who are not in need of any immediate medical attention, but need to undergo a periodic health checkup as a preventive measure

99461	The provider evaluates and manages the care of a normal newborn infant, typically immediately after birth, at a location other than a hospital or birthing center.
99383	The provider performs a well–patient visit for a child who is between the ages of 5 and 11
99392	Preventive medicine services are provided to individuals who are not in need of any immediate medical attention but need to undergo a periodic health checkup as a preventive measure.
99382	The provider performs a well–patient visit for a child who is between the ages of 1 and 4 who meets the definition of new patient.

Supplemental Table S2: Vaccination CPT Codes.

Childhood Vaccinations	CPT Code	Description
DTaP	90698	The provider administers a combination vaccine intramuscularly to protect a patient against five serious diseases: diphtheria, tetanus, pertussis, or whooping cough, *Haemophilus influenzae* type B infections and poliomyelitis, or polio.
	90700	The provider administers a combination vaccine intramuscularly for diphtheria, tetanus toxoids, and acellular pertussis to individual younger than seven years.
	90723	The provider administers a combination inactivated vaccine intramuscularly to prevent multiple diseases, including diphtheria, tetanus, acellular pertussis, hepatitis B, and polio.
IPV	90698	The provider administers a combination vaccine intramuscularly to protect a patient against five serious diseases: diphtheria, tetanus, pertussis, or whooping cough, *Haemophilus influenzae* type B infections and poliomyelitis, or polio.
	90713	The provider administers an inactivated poliovirus vaccine either subcutaneously or intramuscularly to prevent the patient from getting polio.
	90723	The provider administers a combination inactivated vaccine intramuscularly to prevent multiple diseases, including diphtheria, tetanus, acellular pertussis, hepatitis B, and polio.
Measles	90710	The provider administers a live combination vaccine for measles, mumps, varicella and rubella virus subcutaneously to prevent these diseases.
MMR (measles, mumps, rubella)	90707	The provider administers a live combination vaccine for measles, mumps, varicella and rubella virus subcutaneously to prevent these diseases.

HIB	90644	In this procedure, the provider administers a combination vaccine that protects against specific strains of meningococcus and *Haemophilus influenzae* type B, bacteria that cause meningitis, a serious disease that attacks the meninges, the covering of the brain and spinal cord. He injects the vaccine into a muscle in an infant between 6 weeks and 18 months of age as part of a multiple dose schedule.
	90647	In this procedure, the provider administers a form of the vaccine that protects against *Haemophilus influenzae* type B, bacteria that causes meningitis, a serious disease that attacks the meninges, the covering of the brain and spinal cord. He injects the vaccine into a muscle as part of a three–dose schedule.
	90648	In this procedure, the provider administers a form of the vaccine that protects against *Haemophilus influenzae* B, bacteria that causes meningitis, a serious disease that attacks the meninges, the covering of the brain and spinal cord. He injects the vaccine into a muscle as part of a four–dose schedule.
	90698	The provider administers a combination vaccine intramuscularly to protect a patient against five serious diseases: diphtheria, tetanus, pertussis, or whooping cough, *Haemophilus influenzae* type B infections and poliomyelitis, or polio.
	90748	The provider administers a combination vaccine for hepatitis B and *Haemophilus influenzae* type B intramuscularly. This vaccine provides protection against hepatitis and influenza.
Hep B	90723	The provider administers a combination inactivated vaccine intramuscularly to prevent multiple diseases, including diphtheria, tetanus, acellular pertussis, hepatitis B, and polio.
	90740	The provider administers the hepatitis B vaccine intramuscularly in three separate doses to an immunosuppressed patient or a patient undergoing dialysis.
	90744	The provider administers hepatitis B vaccine intramuscularly in three separate doses to a pediatric or adolescent patient to prevent hepatitis B in the liver.
	90747	The provider uses this vaccine to prevent the hepatitis B virus or HBV infection, a viral infection of the liver.
	90748	The provider administers a combination vaccine for hepatitis B and *Haemophilus influenzae* type B intramuscularly. This vaccine provides protection against hepatitis and influenza.
VZV	90710	The provider administers a live combination vaccine for measles, mumps, varicella and rubella virus subcutaneously to prevent these diseases.
	90716	The provider administers a live vaccine subcutaneously to protect against varicella infection.
PCV	90670	In this procedure, the provider administers a vaccine that protects infants and young children against 13 specific strains of pneumococcus, a bacterial organism that causes ear infections, pneumonia, and other infectious diseases, including meningitis, a disease that attacks the meninges, the covering of the brain and spinal cord.

Hep A	90633	The provider injects an altered form of the hepatitis A virus into a muscle in a child or teenager to provide immunity to hepatitis A, an infectious disease of the liver, as part of a two-dose schedule
RV Rotarix	90681	The provider administers two doses of live attenuated rotavirus vaccine by mouth to prevent rotavirus related gastroenteritis with vomiting and diarrhea.
RV RotaTeq	90680	The provider administers three scheduled doses of a live pentavalent rotavirus vaccine by mouth to prevent rotavirus related gastroenteritis that causes vomiting and diarrhea. Prolonged vomiting and diarrhea can lead to dehydration, especially in an infant.
